# Prevalence, indications and neonatal complications of caesarean deliveries in Cameroon: a systematic review and meta-analysis

**DOI:** 10.1186/s13690-020-00430-1

**Published:** 2020-06-03

**Authors:** Tsi Njim, Bayee Swiri Tanyitiku, Clarence Mbanga

**Affiliations:** 1 Health and Human Development (2HD) Research Group, Douala, Littoral region Cameroon; 2 Higher Institute of Commerce and Management, University of Bamenda, Bamenda, North west region Cameroon; 3 Mankon Sub-divisional Hospital, Bamenda, North west region Cameroon

**Keywords:** Caesarean deliveries, Caesarean sections, Neonatal asphyxia, Cameroon

## Abstract

**Background:**

The trend of increasing caesarean deliveries in developed countries over the past three decades is now being observed in sub-Saharan African. This rise might be associated with an increase in the complications that could arise from this surgical intervention. We therefore sought to assess the prevalence, indications and complications of caesarean deliveries in Cameroon.

**Methods:**

We systematically searched online databases: Medline; Global Health and the CINAHL from 01st January 1966 to 25th July 2019. We reviewed published cohort studies, retrospective register analysis and cross-sectional studies that described cohorts of pregnant women presenting at delivery facilities in Cameroon; and included those that had an estimate of the proportion of women who delivered by caesarean sections.

**Results:**

There were 126 articles initially identified by the search and 88 articles were retained after removal of duplicates. After screening of the titles and abstracts, and full text review, we identified 16 articles describing 22 cohorts of women presenting for delivery in health facilities in Cameroon. The overall estimate for the prevalence of caesarean deliveries was 9.9% (95% CI: 7.4, 12.8%, I^2^ = 99.68%, χ^2^ = 315.9, *p* < 0.001). The prevalence of caesarean deliveries increased progressively from 3.4% (95% CI: 2.2, 4.8%) before the year 2000, to 9.8% (95% CI: 7.4, 12.8%) between 2000 and 2009 and 14.7% (95% CI: 8.8, 21.7%) from 2010 to 2019. The three commonest indications for caesarean deliveries were: cephalopelvic disproportion (27.5%; 95% CI: 17.5, 38.7%); previous caesarean deliveries (13.2%; 95% CI: 7.4, 20.3%) and foetal distress (11.2%; 95% CI: 4.8, 19.5%). Neonates who were born by caesarean delivery were more likely to have neonatal asphyxia when compared with neonates born from vaginal deliveries (OR: 6.5; 95% CI: 2.5, 16.5).

**Conclusion:**

The rates of caesarean deliveries in Cameroon falls just within the recommended 10–15% range proposed by the World Health Organisation but have however been increasing progressively in the past decades. There is a strong need to assess the various indications of caesarean deliveries in Cameroon in order to curb its associated complications.

## Background

A caesarean delivery is a surgical intervention which consists of making an incision on the walls of the abdomen and the uterus, for delivery of the baby. It is one of the most commonly performed surgical interventions globally [1], and it is primarily performed as a live-saving procedure when the health of the mother or baby is deemed to be at significant risk, if a vaginal delivery is attempted [2, 3].

The World Health Organization (WHO) recommends caesarean delivery rates of 10–15% [1, 4, 5]. This is based on evidence from several reports suggesting that at 10–15%, caesarean deliveries are associated with a significant reduction of maternal and perinatal morbidity and mortality [6, 7]. At higher rates, the benefits associated with caesarean deliveries fade away [8, 9], and as is the case with most other surgical procedures, exposes both mother and baby to significant short and long term risks and complications [1, 8, 9].

This is worrisome considering the rise in the rates of caesarean deliveries over the past three decades [3, 10]. This rise is highest in high- and middle-income countries, with countries in Western Europe, North America and South America now registering caesarean delivery rates of over 30% [3, 10–12]. This increase in caesarean delivery rates could partly be explained by the rising number of caesarean sections performed at the request of the mothers due to a perceived relative safety, in both developed and developing countries [3, 13].

An increase in caesarean delivery rates, similar to the trend observed above has been noted in Sub-Saharan Africa, raising serious concern from several authors [3, 14]. These concerns are due to the lack of comprehensive and appropriate obstetric care in health facilities, needed to manage the potential risks and complications that could result from a caesarean delivery, in some of the rural settings in these countries [15–17]. In such settings, estimating the prevalence and indications of caesarean deliveries could help policymakers with strategy implementations. A comprehensive knowledge on the general and setting-specific complications associated with caesarean deliveries could help clinicians anticipate the possible outcomes in both mother and child and guide them in revising or establishing new management plans.

We therefore sought to carry out this review to systematically assess the prevalence, indications and complications of caesarean deliveries in Cameroon.

## Methods

### Setting

Cameroon is a multi-ethnic country made up of ten geopolitical regions with 24 million inhabitants (Additional file 1) [18]. As of 2017, 55.5% of the population were living in an urban area [19]. The ten regions (in order of the regions with the most populated cities) are: Littoral; Centre; Far North; North; West; North west; Adamawa; East; South west and South regions [20] (Additional file 1).

### Study design and eligibility criteria

This review was conducted following a predesigned protocol which was e-registered in the PROSPERO database (Registration number: CRD42019144543); and reported according to the Preferred Reporting Items for Systematic Reviews and Meta-Analyses (PRISMA) guidelines. We systematically searched online databases including Medline; Global Health and the CINAHL databases using the following keywords, search terms and phrases: (“caesarean section”, “caesarean delivery” or “operative delivery” and “Cameroon”); coupled with their associated medical subject headings (MeSH). The search started from 01st January 1966 to 25th July 2019. The search strategy used was produced by an information specialist (Additional file 2). We reviewed published cohort studies, retrospective register analysis and cross-sectional studies that included pregnant women presenting at delivery facilities in Cameroon; and which provided an estimate of the prevalence of caesarean deliveries (Table 1). A grey literature search was also carried out by assessing book chapters and documents from organizations such as WHO, and the United States Centers for Disease Control and Prevention (CDC). We excluded studies that did not report primary data such as letters, systematic reviews and commentaries.

**Table 1:** PICOS strategy for inclusion criteria of studies into review

**PICOS strategy**	**Inclusion criteria**
**P-population**	Pregnant women who present for deliveries across hospitals in Cameroon
**I-intervention/Exposure**	Caesarean delivery
**C-comparison**	Women who have vaginal deliveries or instrumental vaginal deliveries
**O-outcome(s)**	Neonatal complications: asphyxia and stillbirth
**S-study design**	Cross-sectional and cohort studies

### Data management

Articles returned by the search were retrieved electronically and saved to EndNote version × 8 software, which was used to screen and remove duplicates. The titles and abstracts of the remaining articles were screened against the above inclusion and exclusion criteria for eligibility independently by two reviewers – TN & BST. Disagreements were handled by discussion and consensus between the two reviewers.

The full texts of eligible articles were downloaded for data extraction. For articles with missing information necessary for the review, the authors of the article were contacted by email requesting the information.

A tool designed on Microsoft Excel 2010 prior to the search and pretested by the principal investigator – TN, was used for data extraction.

### Data items and extraction

The data extraction tool was used by the two aforementioned independent reviewers to extract the following general information from each article that satisfied the inclusion criteria: Last name of first author; date of publication; region in which the study took place; proportion of participants who gave birth through caesarean deliveries; various indications of caesarean deliveries; the study design; age range of participants; sample size; duration of data collection, time of participant recruitment and various maternal and neonatal complications of caesarean deliveries.

### Assessment of methodological quality and risk of bias

The two independent reviewers used the Quality Assessment Tool for Observational Cohort and Cross-Sectional Studies of the National Health Institute/National Heart, Lung, and Blood Institute to assess for methodological quality (Additional file 3). Studies were deemed of good quality if > 70% of the applicable criteria were attained in the quality assessment tool, fair quality if ≥ 40–70% of the applicable criteria were attained in the quality assessment tool and poor quality if < 40% of the applicable criteria were attained in the quality assessment tool.

### Data synthesis and analysis

A meta-analysis was performed to obtain an overall pooled measure of the proportion of caesarean deliveries in Cameroon with a subgroup analyses done to obtain pooled effects for different groups: regions, time periods (before 2000; between 2000 and 2009 and from 2010 to 2019), settings (rural, semi-urban and urban) and types of health facilities (primary, secondary and tertiary).

The various indications of caesarean deliveries were described, and a meta-analysis performed for each indication if at least two studies listed the indication in their results. A meta-analysis was also performed to assess the relationship between the complications and caesarean deliveries using the odds ratio as the measure of the estimate. Meta-analyses and subgroup analyses were only performed if at least two studies reported the relevant outcome or subgroup.

### Assessment of heterogeneity

Due to the variability of the different study settings and study designs; a random effects meta-analysis model was used for interpretation over fixed-effects models. The χ^2^ test for heterogeneity and the I^2^ statistic were used to assess the degree of heterogeneity.

## Results

**Fig. 1:**
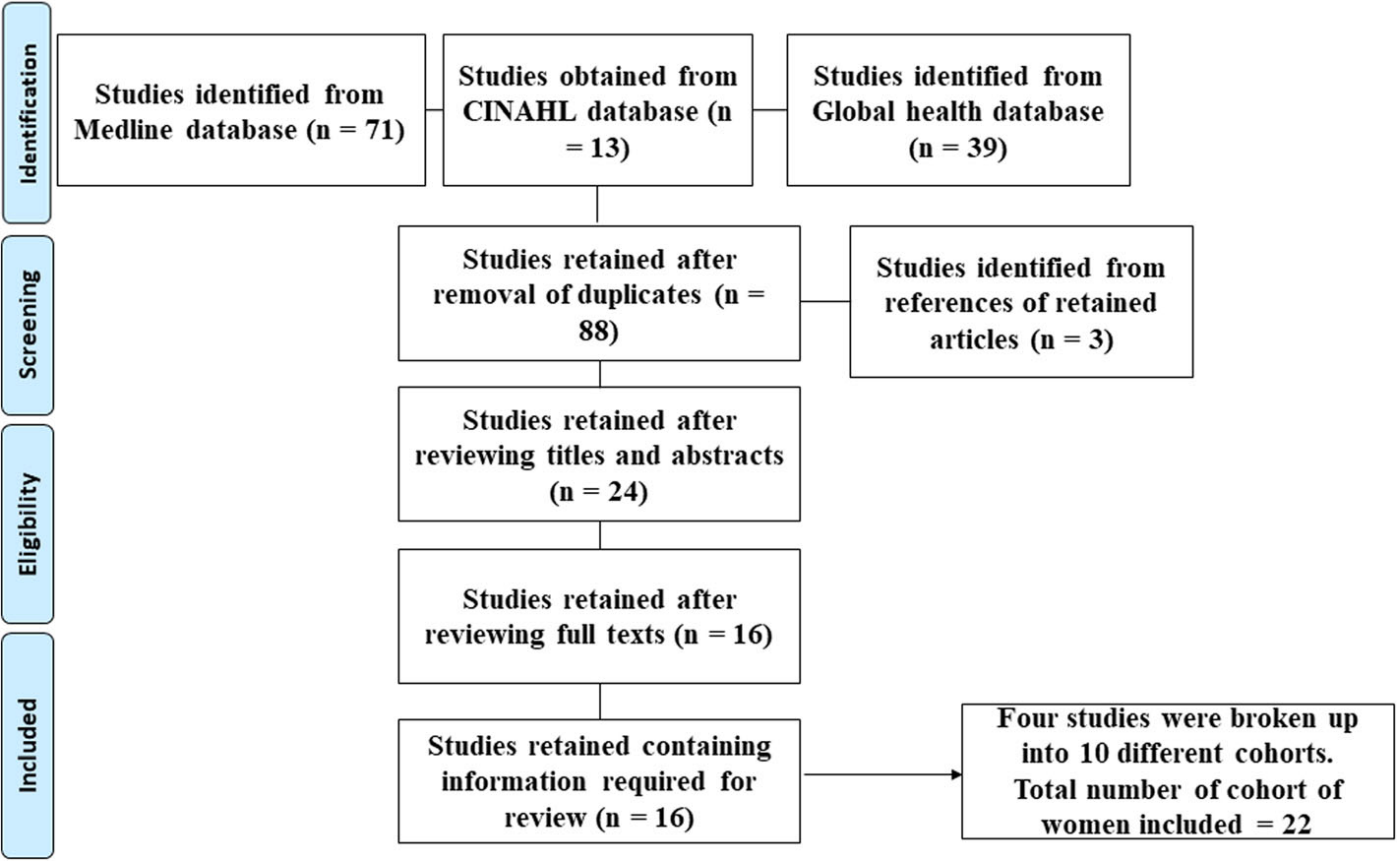
Flow chart showing inclusion and exclusion of studies in the review

There were 123 articles initially identified by the search (Fig. 1). Eighty-eight articles were retained after removal of duplicates. Three articles were further identified from the references of the articles retained from the search. After screening of the titles and abstracts, 24 articles were retained for full text review. The full text review identified 16 articles that reported on the prevalence of caesarean deliveries in Cameroon.

Three of the studies could be broken up into two separate cohorts of women each [3, 21, 22], while one of the studies (the Demographic Health survey analysis) was broken up into four cohorts of women [23]. The other studies each reported on a single cohort of women [22, 24–35]. In total, 22 cohorts of women were included in the review. These cohorts included a total of 269,006 women presenting for deliveries in health facilities. The characteristics of the cohorts of pregnant women included in the review are summarised in Table 2.

**Table 2:** Characteristics of studies included in the review

**Author name**	**Publication year**	**Years of patient recruitment**	**Type of study**	**Region**	**Age profile**	**Type of health facility**	**Setting**	**sample size**	**Number with CD**	**Complications**^**a**^	**Indications**
Ahounkeng [25]	2014	2011	Cross-sectional	Centre	16–43	Tertiary	Urban	231	43	No	No
DHS-1a [23]	2003	1991	Cross-sectional	Not specified	NC	Not specified	Urban	24,182	798	No	No
DHS-1b [23]	2003	1991	Cross-sectional	Not specified	NC	Not specified	Rural	72,824	1238	No	No
DHS-1c [23]	2003	1998	Cross-sectional	Not specified	NC	Not specified	Urban	18,485	610	No	No
DHS-1d [23]	2003	1998	Cross-sectional	Not specified	NC	Not specified	Rural	72,391	1665	No	No
Njim-1a [3]	2017	2007–2012	Cross-sectional	South west	26.4 ± 5.5	Secondary	Semi-urban	4941	1006	No	No
Njim-1b [3]	2017	2013	Cross-sectional	South west	26.4 ± 5.8	Secondary	Semi-urban	200	25	Yes	Yes
Tanyi [34]	2016	2015	Cross-sectional	South west	15–44	Secondary	Semi-urban	1492	199	Yes	Yes
Tebeu [35]	2008	2003–2004	Cross-sectional	Far north	NC	Secondary	Semi-urban	3263	144	Yes	Yes
Foumane [29]	2014	2012	Cohort	Centre	NC	Tertiary	Urban	1108	219	Yes	Yes
Ngowa [30]	2015	2012	Cohort	Centre	28.1 ± 0.9	Tertiary	Urban	2330	460	No	No
Mbassi-1a [21]	2011	2005–2006	Cross-sectional	Centre	NC	Tertiary	Urban	22,842	2691	No	No
Mbassi-1b [21]	2011	2005–2006	Cross-sectional	Littoral	NC	Tertiary	Urban	12,056	1378	No	No
Fouelifack [28]	2014	2008–2010	Cross-sectional	Centre	27.34 ± 6.03	Tertiary	Urban	5921	876	No	No
Fouelifack [27]	2015	2014	Cross-sectional	Centre	27.6 ± 5.9	Tertiary	Urban	462	176	No	No
Doh [26]	1991	1982–1989	Cross-sectional	Centre	NC	Tertiary	Urban	9637	741	Yes	Yes
Nana-1a [22]	2011	2007	Cross-sectional	Far north	15–40	Secondary	Semi-urban	1070	61	Yes	Yes
Nana-1b [22]	2011	2007	Cross-sectional	Far north	15–40	Secondary	Rural	484	30	Yes	Yes
Nkwabong [32]	2011	2007–2008	Cross-sectional	Centre	16–38	Tertiary	Urban	4342	719	No	No
Njim-2 [31]	2017	2015–2016	Cross-sectional	North west	26.6 ± 5.3	Secondary	Semi-urban	886	109	No	No
Tamambang [33]	2018	2010–2015	Cross-sectional	Littoral	26.6 ± 6.4	Tertiary	Urban	8056	495	No	No
Agbor [24]	2017	2009–2016	Cross-sectional	North west	26.0 ± 6.5	Primary	Rural	1803	16	No	No

*CD* caesarean deliveries, *NC* Not clear, ^a^ Complications included in meta-analysis

Two of the cohorts were from the North west region [24, 31], two were from the Littoral region [21, 33], eight from the Centre region [21, 25–30, 32], three from the South west region [3, 34], three from the Far north region [22, 35] and the Demographic Health survey analysis did not specify regions [23]. All the studies used a cross-sectional design except Ngowa et al and Foumane et al [29, 30] which utilised a cohort design and Tebeu et al [35] used an additional case-control design to assess the complications of caesarean deliveries.

Four of the cohorts described pregnant women in rural areas [22–24], six in semi-urban areas [3, 22, 31, 34, 35] and twelve in urban areas [21, 23, 25–30, 32, 33]. The studies were also divided into three time periods depending on when participants were recruited – before 2000 [23, 26]; between 2000 and 2009 [21, 22, 28, 32, 35] and from 2010 to 2019 [3, 25, 27, 29–31, 33, 34]. Seven of the cohorts had their deliveries in secondary health facilities [3, 22, 31, 34, 35], ten in tertiary hospitals and one in a primary health care facility [24].

The Quality Assessment Tools for observational studies of the National Health Institute/National Heart, Lung, and Blood Institute was used to assess methodological quality. Twelve of the articles were of “good quality” [3, 22, 24–31, 33, 34]; three were of “fair quality” [21, 32, 35] and one was of “poor quality” [23] (Additional file 3).

### Prevalence of caesarean deliveries

**Fig. 2:**
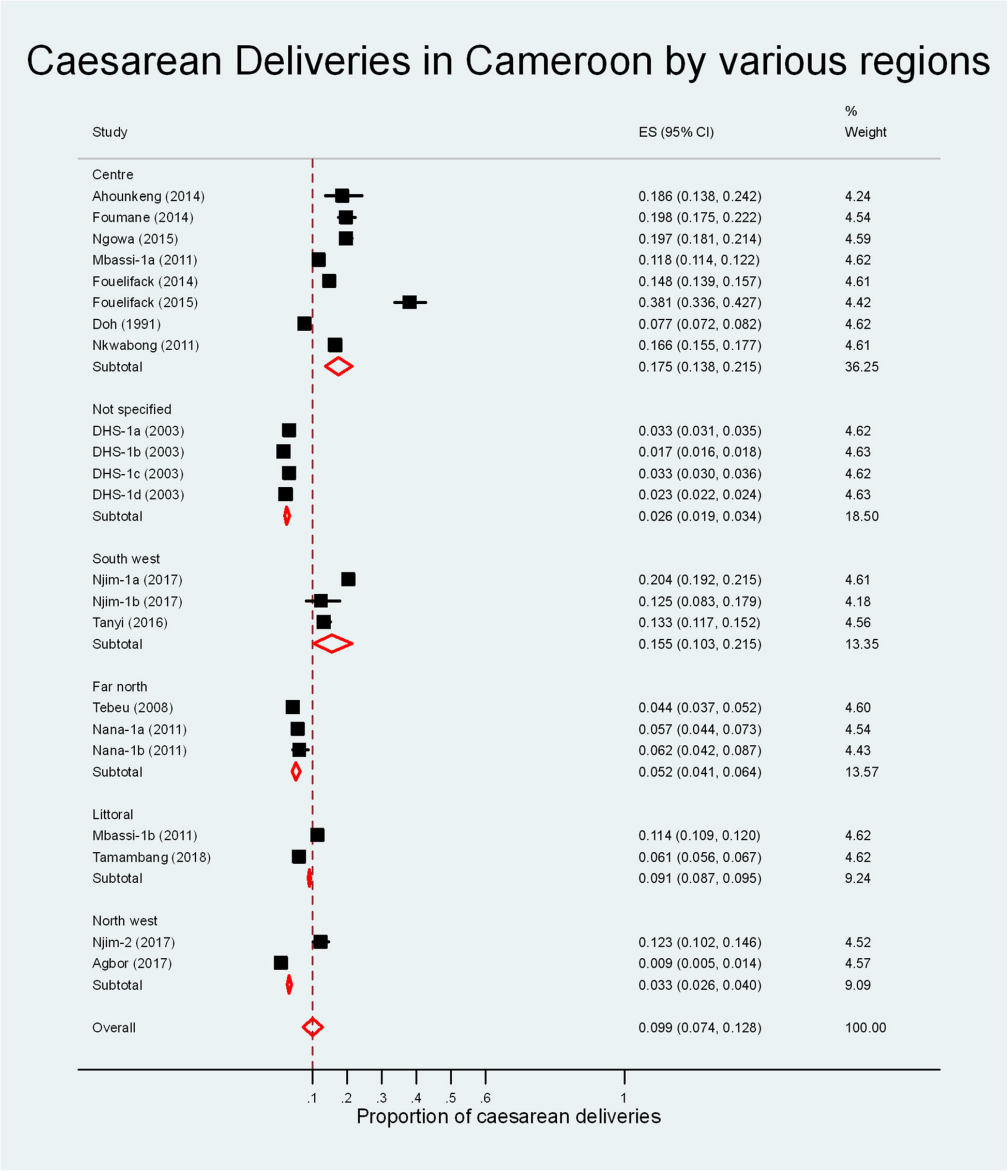
Meta-analyis of the proportion of caesarean deliveries in Cameroon

The overall estimate for the prevalence of caesarean deliveries in Cameroon was 9.9% (95% CI: 7.4, 12.8%, I^2^ = 99.68%, χ^2^ = 315.9, *p* < 0.001). Figure 2 shows the pooled prevalence for the various regions in the country. The Centre region had the highest prevalence of caesarean deliveries 17.5% (95% CI: 13.8, 21.5%) while North west region had the lowest prevalence at 3.3% (95% CI: 2.6, 4.0%).

The prevalence of caesarean deliveries increased progressively throughout the time periods from 3.4% (95% CI: 2.2, 4.8%) before the year 2000, to 9.8% (95% CI: 7.4, 12.5%) between 2000 and 2009 and 14.7% (95% CI: 8.8, 21.7%) from 2010 to 2019 (Additional file 4).

The prevalence of caesarean deliveries in rural areas – 2.1% (95% CI: 1.5, 2.7%) was lower than that in semi-urban areas – 10.8% (95% CI: 5.2, 18.2%) and urban areas – 12.9% (95% CI: 9.3, 17.0%) (overall I^2^ = 99.8%, χ^2^ = 10,391.0, *p* < 0.001) (Additional file 4).

The prevalence of caesarean deliveries in secondary health facilities around the country – 10.1% (95% CI: 5.1, 16.6%) was similar to that in tertiary hospitals – 15.4% (95% CI: 12.5, 18.6%); overall I^2^ = 99.8%, χ^2^ = 10,391.0, p < 0.001 (Additional file 4).

### Indications of caesarean deliveries in Cameroon

Seven of the cohorts had data on the various indications of caesarean deliveries [3, 22, 26, 29, 34, 35]. Table 3 shows the various indications of caesarean deliveries and their frequencies as found in each individual study. At least two studies reported a frequency for eight of the indications (Additional file 5): cephalopelvic disproportion (27.5%; 95% CI: 17.5, 38.7%) [3, 22, 26, 29, 34, 35]; previous caesarean section (13.2%; 95% CI: 7.4, 20.3%) [3, 22, 26, 29, 34, 35]; foetal distress (11.2%; 95% CI: 4.8, 19.5%) [3, 22, 26, 29, 34, 35]; malpresentation (9.8%; 95% CI: 7.0, 12.9%) [3, 26, 29, 34, 35]; antepartum haemorrhage (8.2%; 95% CI: 5.9, 10.8%) [26, 29, 34, 35]; dystocia (5.9%; 95% CI: 2.0, 11.3%) [3, 26, 29, 35]; multiple pregnancies (5.8%; 95% CI: 2.0, 11.1%) [22, 29, 34, 35]; macrosomia (5.7%; 95% CI: 3.7, 8.0%) [22, 29, 34]; cord prolapse (4.0%; 95% CI: 1.9, 6.8%) [26, 29, 35]; hypertensive disorders in pregnancy (3.1%; 95% CI: 2.1, 4.2%) [26, 34, 35] and uterine rupture (2.6%; 95% CI: 0.6, 5.5%) [26, 29, 35].

**Table 3:** Various indications of caesarean deliveries and their relative frequencies as reported in the studies

Author name	sample size (N)	Indications of CD
Njim-1b, 2017 [3]	200	Cephalopelvic disproportion (7/25), acute foetal distress (7/25), previous CS (4/25), malpresentation (3/25), maternal request (3/25), dystocia (1/25)
Tanyi, 2016 [34]	1492	Cephalopelvic disproportion (64/199), previous CS (55), malpresentation (26/199), foetal distress (16), foetal macrosomia (15), placenta praevia (9), HTN disorders (6), multiple pregnancy (2), placenta abruption (1)
Tebeu, 2008 [35]	3263	Cephalopelvic disproportion (47/144), placenta previa (13/144), cord prolapse (10/144), uterine rupture (9/144), arm prolapse (7/144), malpresentation (7/144), fibroid previa (2/144), placenta abruption (2/144), multiple pregnancy (10/144), dystocia (6/144), hypertensive disorders (4/144), previous CS (3/144), malformation (3/144), foetal distress (2/144), undefined (19/144)
Foumane, 2014 [29]	1108	Cephalopelvic disproportion (21/219), previous CS (33/219), antepartum haemorrhage (24/219), malpresentation (20/219), acute foetal distress (18/219), dystocia (28/219), multiple pregnancy (13/219), macrosomia (11/219), cord prolapse (9/219), old primipa (7/219), uterine rupture (4/219), PMTCT (3/219), DVT (2/219)
Doh, 1991 [26]	9637	Cephalopelvic disproportion (109/741), malpresentation (88/741), foetal distress (159/741), antepartum haemorrhage (57/741), cord prolapse (19/741), hypertensive disorders (24/741), uterine rupture (10/741), previous CS (94/741), failed induction/dystocia (29/741), others (152/741)
Nana-1a, 2011 [22]	1070	Cephalopelvic disproportion (28/61), foetal distress (7/61), multiple pregnancy (9/61), previous CS (8/61), macrosomia (2/61), others (7/61)
Nana-1b, 2011 [22]	484	Cephalopelvic disproportion (13/30), foetal distress (4/40), multiple pregnancy (2/30), previous CS (4/30), others (7/30)

*N* total number of pregnant women, *HTN* hypertension, *CS* caesarean section, *PMTCT* prevention of mother to child transmission, *DVT* deep vein thrombosis

### Complications of caesarean deliveries

Only two studies compared the complications of neonates from caesarean deliveries with neonates from vaginal deliveries [3, 35] (Additional file 6).

Neonates who were born by caesarean delivery were more likely to have neonatal asphyxia when compared with neonates born from vaginal deliveries (OR: 6.5; 95% CI: 2.5, 16.5; I^2^ = 0.0%, χ^2^ = 0.5, *p* = 0.5) while neonates born from a caesarean delivery were just as likely to be stillborn as neonates born from vaginal deliveries (OR: 3.5; 95% CI: 0.0, 1,348,755.5; I^2^ = 96.0%, χ^2^ = 23.5, *p* < 0.01).

### Conceptual framework

**Fig. 3:**
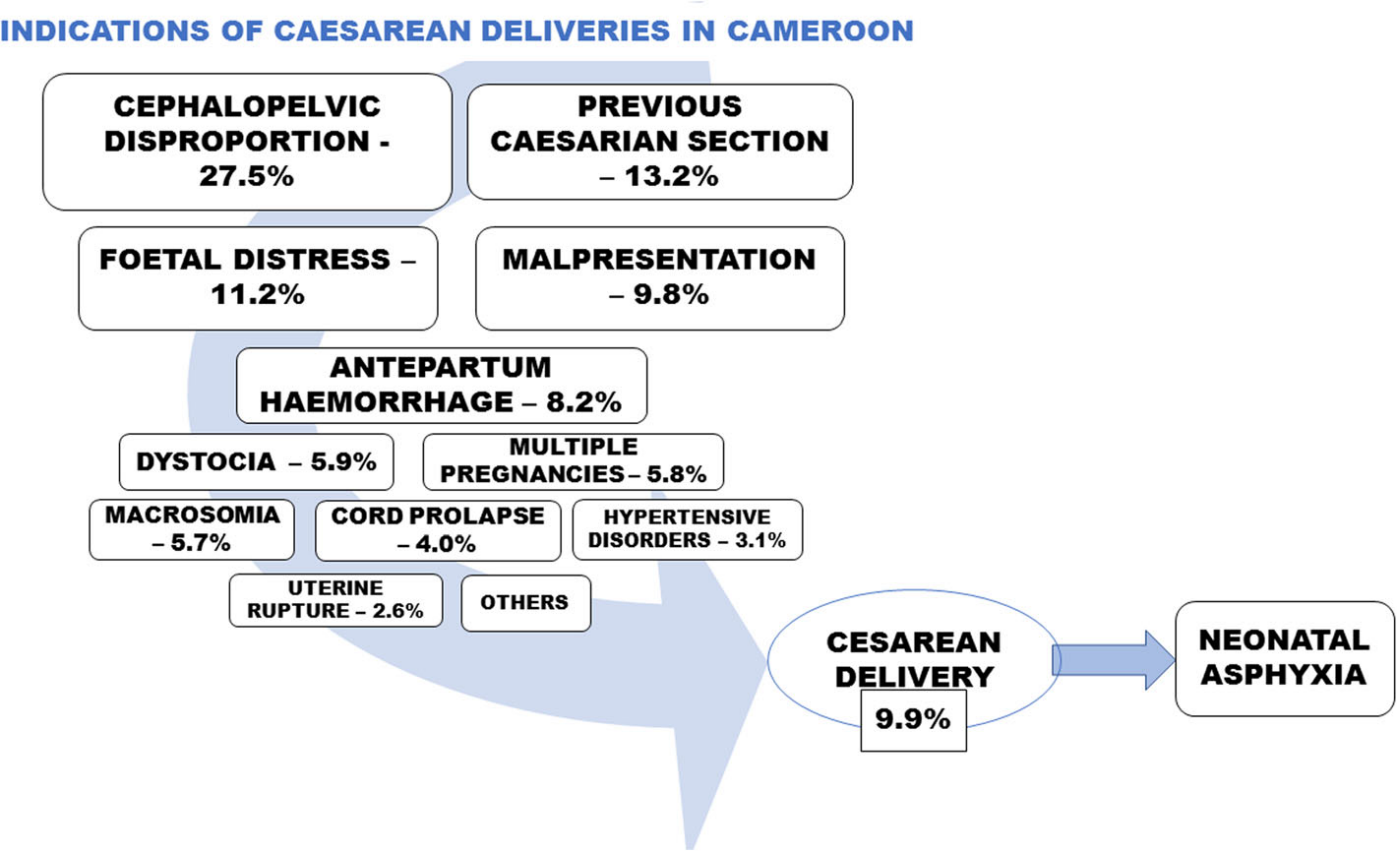
Conceptual framework of the indications and complications of caesarean deliveries in Cameroon

The conceptual framework summarising the indications and complications of caesarean deliveries in Cameroon is shown in Fig. 3.

## Discussion

In this review, the overall prevalence of caesarean sections in Cameroon was 9.9%. The prevalence ranged from 3.3% in the North west region to 17.5% in the centre region and increased progressively over the years from 3.4% prior to 2000 to 14.7% after the year 2010. The most common indications of caesarean sections in the country were cephalopelvic disproportion, previous caesarean sections and foetal distress; while neonates who were born by caesarean sections were significantly more likely to have neonatal asphyxia at birth.

From the pooled overall prevalence (and 95% confidence limits) of caesarean deliveries in Cameroon, it seems to fall just within the recommended WHO range of 10–15% [4, 5]. Some regions like the North west region (3.3%) and the Far north region (5.2%) fell well below this range. Other regions like the South west region (15.5%) were just within the target while the prevalence was much higher in the centre region (17.5%). This could be explained by the fact that the Centre region has more tertiary health facilities than the other regions in the country (Additional file 1). The tertiary health facilities in the country are at the top of the referral chain and hence carry out more emergency procedures (caesarean sections inclusive) than the secondary and primary health facilities. Indeed, our review showed that tertiary health facilities carried out more caesarean sections (15.4%) than secondary health facilities (10.1%). This assertion is supported by the study carried out by Agbor et al in primary health facilities of a rural area of the North west region of Cameroon where the low rates of caesarean deliveries (0.9%) were explained by the absence of a permanent doctor in these facilities [24].

The rates of caesarean sections in Cameroon were however lower when compared with other countries in Africa like Ethiopia (27.6%), Libya (23.5%) and Rwanda (64.2%) [36–38]. The rates were also lower than those in Asian and Middle-eastern countries: Pakistan (24.1%), Iran (48%), India (13.7–37.9%) [39–41]; South America: Brazil (55.5%) [42] and Europe: Cyprus (52.2%) and Iceland (14.8%) [43]. These high caesarean section rates are ever increasing worldwide [3, 10], even in sub-Saharan African countries [3, 14]. This trend was also shown to be true in Cameroon as demonstrated in this review. The prevalence of caesarean deliveries increased from 3.4% before the year 2000, to 9.8% between 2000 and 2009 and 14.7% from 2010 to 2019. With this trend, it could be estimated that the rates of caesarean deliveries in Cameroon will soon surpass the WHO recommended range. This therefore points to the need for investigations into factors driving this trend and harmonisation of obstetric care around the country.

The three main indications of caesarean deliveries seen in this review were cephalopelvic disproportion, a previous caesarean section and foetal distress. The above indications were found to be the commonest indications for caesarean births in Ethiopia [36]. These three indications were also found to be in the top five commonest medical factors driving the decision in performing a caesarean section in a review performed in India [40]. The authors showed that previous caesarean sections, foetal distress, failure of labour progression, cephalopelvic disproportion, maternal disease and abnormal presentation were the most common causes of caesarean deliveries [40]. Similarly, in Pakistan, Najmi et al determined that repeat caesarean section and foetal distress were amongst the commonest indications for caesarean deliveries in a tertiary hospital [39]. Considering that these indications are universal, this could be useful information for healthcare providers especially in rural areas in Cameroon where caesarean sections are not routinely performed [24]. Women presenting with the following indications at delivery should be considered as high-risk deliveries and referred appropriately to services where caesarean sections could easily be performed. This could help to reduce maternal and perinatal mortality and morbidity associated with childbirth in the country.

In this review, neonates who were born by caesarean delivery were also more likely to have neonatal asphyxia when compared with neonates who were born through vaginal deliveries. Considering that most of the indications of caesarean sections obtained in this review – foetal distress, dystocia, cord prolapse and antepartum haemorrhage; include obstruction of blood and oxygen supply to the foetus, it is therefore conceivable that neonates who are born through caesarean sections will be more likely to have asphyxia at birth. Also, in Cameroon, most cases requiring obstetrical care are first received at the primary health care facilities. These facilities generally lack both the technical plateau and skilled human resources needed to diagnose the aforementioned complications or perform caesarean deliveries [24]. They therefore end up being referred to facilities higher up the referral chain. However, with the poor distribution of tertiary hospitals around the country, a lot of time is used up to cover significant distances needed to get to these referral centres. Consequently, patients arrive and are managed in an emergency setting. Emergency caesarean deliveries have been shown to be associated with poor perinatal outcome [44].

This highlights the need for delivery services in healthcare facilities around the country to be equipped with both skilled personnel and the necessary resources to diagnose and manage these conditions and perform caesarean sections. Likewise, centres where caesarean deliveries are performed, and physicians, should be ready to anticipate birth asphyxia as a possible caesarean delivery outcome and prepare guidelines and management plans accordingly.

We would like to note some of the limitations of this review. First, five regions in the country did not have separate data providing the rates of caesarean sections in this region. The overall estimate provided in this review may therefore underestimate or overestimate the prevalence of caesarean sections. However, the DHS studies included in the review draw from approximately all the regions in the country and could help limit this bias. Secondly, the two studies that measured the association between neonatal asphyxia and caesarean deliveries used the Apgar scores to diagnose asphyxia. The Apgar score is not the gold standard for the diagnosis of asphyxia. It could however be used to assess the probability of a neonate to have asphyxia especially in settings where diagnostic apparatus is limited like in rural and semi-urban health facilities in Cameroon. Thirdly, there was a high degree of heterogeneity seen in some of the meta-analyses. We speculate that this may be due to the different periods of participant recruitment, settings, study designs, number of studies in the review and health facilities used in the various studies. There was significant heterogeneity between the subgroups and the number of studies in each subgroup were not large enough to draw conclusive results. Caution should therefore be used when interpreting the meta-analysis in this review.

## Conclusion

The rates of caesarean deliveries in Cameroon falls just within the recommended 10–15% range proposed by the WHO. The rates in rural and sub-urban settings in the country fall far below this range due to lack of adequate facilities and healthcare personnel required to carry out these surgeries. There is a strong need to recruit healthcare personnel capable of carrying out these deliveries in areas that are lacking and reinforcing the current workforce in hospitals that are already carrying out caesarean deliveries. Also, the commonest indications and complications of caesarean deliveries should be anticipated in women of childbearing age to improve management plans and guidelines and decrease the associated maternal and foetal morbidity and mortality.

## Supplementary information

**Additional file 1.**

**Additional file 2.**

**Additional file 3.**

**Additional file 4.**

**Additional file 5.**

**Additional file 6.**

## Data Availability

Not applicable.

## Abbreviations

OR: Odds ratio
PRISMA: Preferred Reporting Items for Systematic Reviews and Meta-Analyses
