# Supplementary material for: Prevalence, indications and neonatal complications of caesarean deliveries in Cameroon: a systematic review and meta-analysis
Source: Arch Public Health. 2020 Jun 3;78:51. doi: 10.1186/s13690-020-00430-1 (PMC7268214; doi:10.1186/s13690-020-00430-1)
Supplement: Supplementary file 1 — Additional file 1. [file 13690_2020_430_MOESM1_ESM.pdf]

Caesarean deliveries in Cameroon: Setting and context

The most populated cities in each region in the country are: Littoral (Douala - 1,338,082, Edea - 203,149, Loum - 177,429); Centre (Yaoundé - 1,299,369); Far North (Kousseri - 435,547, Maroua - 319,941, Mokolo - 275,239); North (Garoua - 436,899, Guider - 84,647); West (Bafoussam - 290,768, Nkongsamba - 117,063, Mbouda - 111,320, Dschang - 96,112, Foumban - 92,673, Foumbot - 84,065) North west (Bamenda - 393,835); Adamawa (Ngaoundere - 231,357); East (Bertoua - 218,111); South west (Kumba - 144,413) and South (Ebolowa - 87,875) [1].

The figure below shows the population density of Cameroon with respect to the level of urbanization of the various regions and the number of public healthcare tertiary facilities in each of these regions.

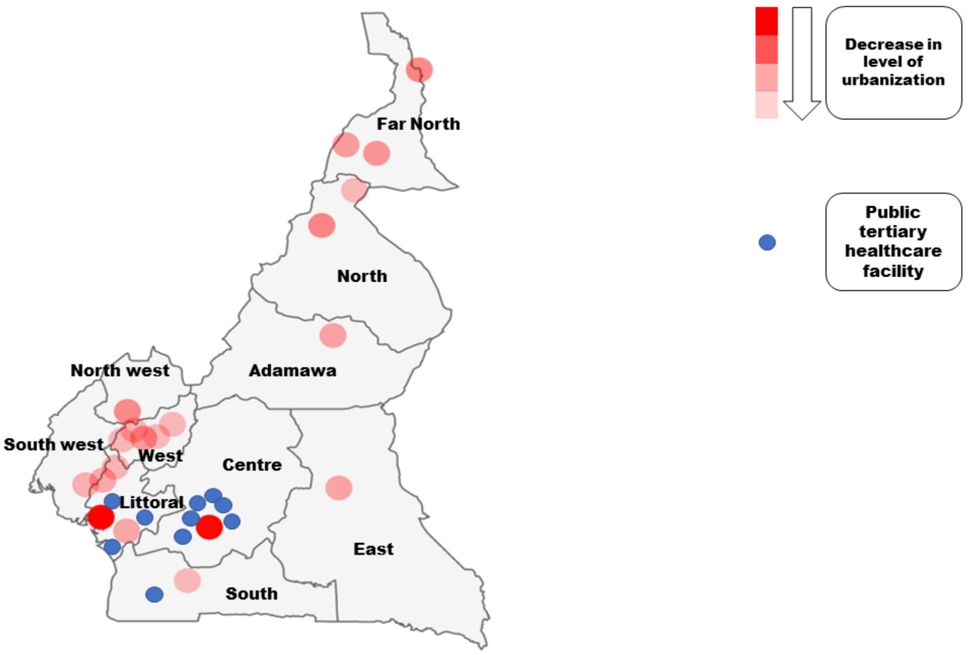

Adapted from [1] - Cameroon Population 2019. Access Date: 30/07/2019. Available from: http://worldpopulationreview.com/countries/cameroon-population/#popDensityMap

*Not drawn to scale.

References:

1. Cameroon Population 2019. Access Date: 30/07/2019. Available from: http://worldpopulationreview.com/countries/cameroon-population/#popDensityMap.
