# Supplementary material for: Prevalence, indications and neonatal complications of caesarean deliveries in Cameroon: a systematic review and meta-analysis
Source: Arch Public Health. 2020 Jun 3;78:51. doi: 10.1186/s13690-020-00430-1 (PMC7268214; doi:10.1186/s13690-020-00430-1)
Supplement: Supplementary file 2 — Additional file 2. [file 13690_2020_430_MOESM2_ESM.pdf]

Information sources

Electronic sources

Online database: Medline was searched using the search strategy below from the date of creation of the database to 25/07/2019, for relevant abstracts of articles.

Searches	Search combinations	Search terms	Number of hits
S1		(MH "Cesarean Section+") OR (MH "Cesarean Section, Repeat")	43,084
S2		“caesarean section*” OR “cesarean section*” OR “caesarean deliver*” OR “cesarean deliver*” OR “operative deliver*” OR “abdominal deliver*” OR “C-section”	68,777
S3	S1 OR S2		68,777
S4		“MH Cameroon+”	5,066
S5		Cameroon	9,912
S6	S4 OR S5		9,912
S7	S3 AND S6		71

Online database: CINAHL was searched using the search strategy below from the date of creation of the database to 25/07/2019, for relevant abstracts of articles.

Searches	Search combinations	Search terms	Number of hits
S1		(MH "Cesarean Section, Elective") OR (MH "Cesarean Section+") OR (MH "Cesarean Section, Repeat")	16,189
S2		“caesarean section*” OR “cesarean section*” OR “caesarean deliver*” OR “cesarean deliver*” OR “operative deliver*” OR “abdominal deliver*” OR “C-section”	22,471
S3	S1 OR S2		22,471
S4		“MH Cameroon+”	1,025
S5		Cameroon	1,278
S6	S4 OR S5		1,278

S7	S3 AND S6		13

Online database: Global health was searched using the search strategy below from the date of creation of the database to 25/07/2019, for relevant abstracts of articles.

Searches	Search combinations	Search terms	Number of hits
S1		"caesarean section*" OR "cesarean section*" OR "caesarean deliver*" OR "cesarean deliver*" OR "operative deliver*" OR "abdominal deliver*" OR "C-section"	7,399
S2		Cameroon	8,741
S3	S1 AND S2		39
