# Supplementary material for: Prevalence, indications and neonatal complications of caesarean deliveries in Cameroon: a systematic review and meta-analysis
Source: Arch Public Health. 2020 Jun 3;78:51. doi: 10.1186/s13690-020-00430-1 (PMC7268214; doi:10.1186/s13690-020-00430-1)
Supplement: Supplementary file 3 — Additional file 3. [file 13690_2020_430_MOESM3_ESM.pdf]

Quality Assessment Tool for Observational Cohort and Cross-Sectional Studies of the National Health Institute/National Heart, Lung, and Blood Institute

	**Criteria**
1	Was the research question or objective in this paper clearly stated?
2	Was the study population clearly specified and defined?
3	Was the participation rate of eligible persons at least 50%?
4	Were all the subjects selected or recruited from the same or similar populations (including the same time period)? Were inclusion and exclusion criteria for being in the study prespecified and applied uniformly to all participants?
5	Was a sample size justification, power description, or variance and effect estimates provided?
6	For the analyses in this paper, were the exposure(s) of interest measured prior to the outcome(s) being measured?
7	Was the timeframe sufficient so that one could reasonably expect to see an association between exposure and outcome if it existed?
8	For exposures that can vary in amount or level, did the study examine different levels of the exposure as related to the outcome (e.g., categories of exposure, or exposure measured as continuous variable)?
9	Were the exposure measures (independent variables) clearly defined, valid, reliable, and implemented consistently across all study participants?
10	Was the exposure(s) assessed more than once over time?
11	Were the outcome measures (dependent variables) clearly defined, valid, reliable, and implemented consistently across all study participants?
12	Were the outcome assessors blinded to the exposure status of participants?
13	Was loss to follow-up after baseline 20% or less?
14	Were key potential confounding variables measured and adjusted statistically for their impact on the relationship between exposure(s) and outcome(s)?
	Quality rating
	Rater #1 TN
	Rater #2 BST

Study	1	2	3	4	5	6	7	8	9	10	11	12	13	14	Rating
Agbor, 2017	Yes	Yes	Yes	Yes	No	Yes	Yes	Yes	Yes	No	Yes	No	Yes	Yes	Good
Ahounkeng, 2014	Yes	Yes	Yes	Yes	No	Yes	Yes	Yes	Yes	No	Yes	No	Yes	No	Good
Buekens, 2003	Yes	Yes	NC	Yes	No	NC	NC	NC	NC	NC	Yes	No	Yes	NC	Poor
Doh, 1991	Yes	Yes	Yes	Yes	No	Yes	Yes	Yes	Yes	No	Yes	No	Yes	Yes	Good
Fouelifack, 2015	Yes	Yes	Yes	Yes	No	Yes	Yes	Yes	Yes	No	Yes	No	Yes	Yes	Good
Fouelifack, 2014	Yes	Yes	Yes	Yes	Yes	Yes	Yes	Yes	Yes	No	Yes	No	Yes	Yes	Good
Foumane, 2014	Yes	Yes	Yes	Yes	No	Yes	Yes	Yes	Yes	No	Yes	No	Yes	Yes	Good
Mbassi, 2011	Yes	Yes	Yes	Yes	No	No	NA	NA	NA	NA	Yes	No	Yes	No	Fair
Nana, 2011	Yes	Yes	Yes	Yes	Yes	Yes	Yes	Yes	Yes	No	Yes	No	Yes	Yes	Good
Ngowa, 2015	Yes	Yes	Yes	Yes	No	Yes	Yes	Yes	Yes	Yes	Yes	No	Yes	No	Good
Njim-2, 2017	Yes	Yes	Yes	Yes	No	Yes	Yes	Yes	Yes	Yes	Yes	No	Yes	Yes	Good
Njim-1, 2017	Yes	Yes	Yes	Yes	No	Yes	Yes	Yes	Yes	Yes	Yes	No	Yes	Yes	Good
Nkwabong, 2011	Yes	Yes	Yes	Yes	No	No	No	No	No	No	Yes	No	Yes	No	Fair
Tamambang, 2018	Yes	Yes	Yes	Yes	No	Yes	Yes	Yes	Yes	Yes	Yes	No	Yes	Yes	Good
Tanyi, 2016	Yes	Yes	Yes	Yes	No	Yes	Yes	Yes	Yes	Yes	Yes	No	Yes	No	Good
Tebeu, 2008	Yes	Yes	Yes	Yes	No	No	No	No	No	No	Yes	No	Yes	No	Fair
