# Supplementary material for: Prevalence, indications and neonatal complications of caesarean deliveries in Cameroon: a systematic review and meta-analysis
Source: Arch Public Health. 2020 Jun 3;78:51. doi: 10.1186/s13690-020-00430-1 (PMC7268214; doi:10.1186/s13690-020-00430-1)

Meta-analysis showing the prevalence of caesarean deliveries in Cameroon over different time periods, taken from the period of recruitment of the participants – before the year 2000, 2000 – 2009 and 2010 – 2019.

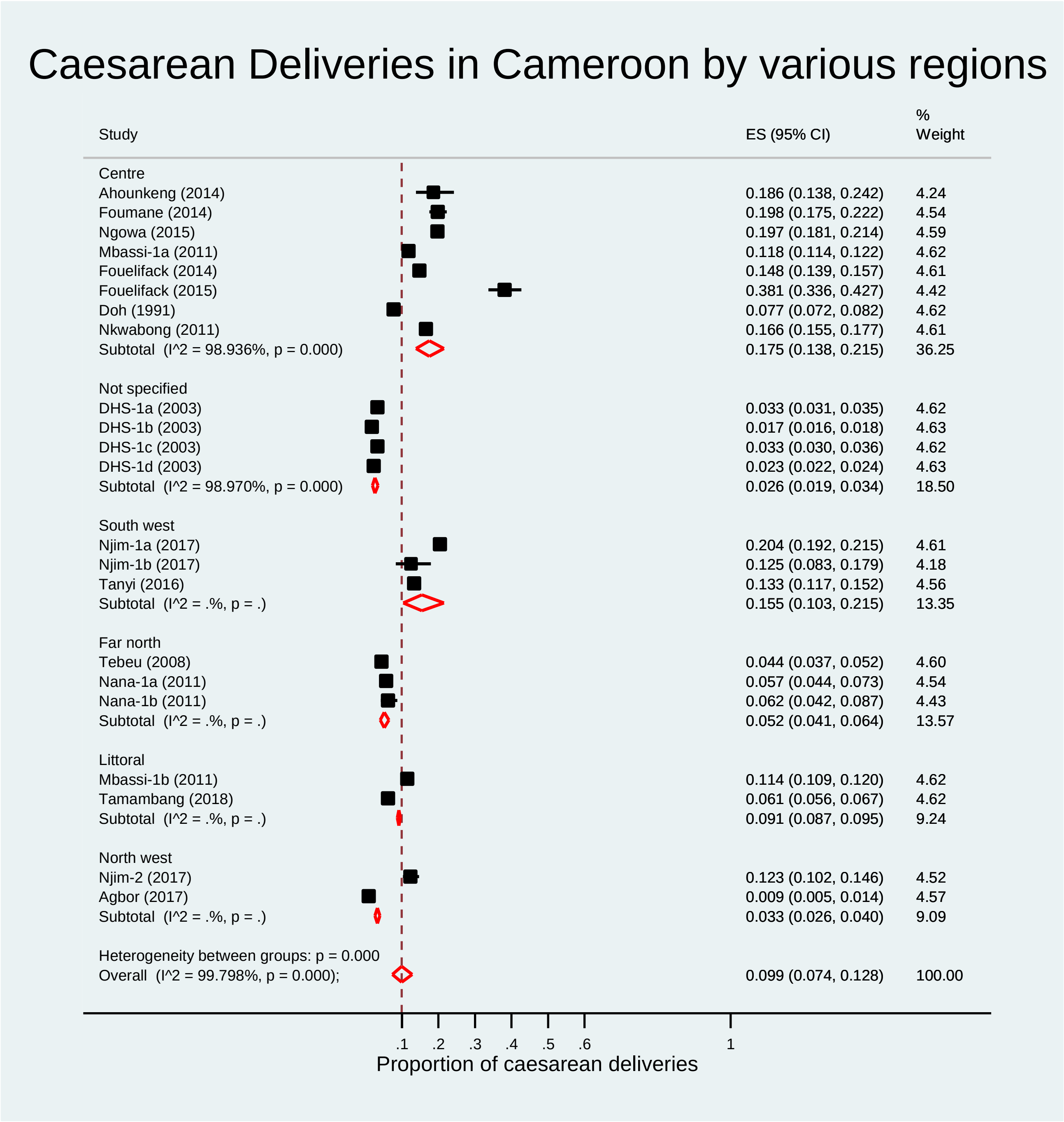

The study Njim-1a et al recruited participants from 2007 – 2012. As this fell between two time periods, it was placed in the time period 2010 – 2019 a majority (52.8%) of the participant recruitment occurred in this period.

Meta-analysis showing the prevalence of caesarean deliveries in Cameroon in different settings – rural, urban and semi-urban.

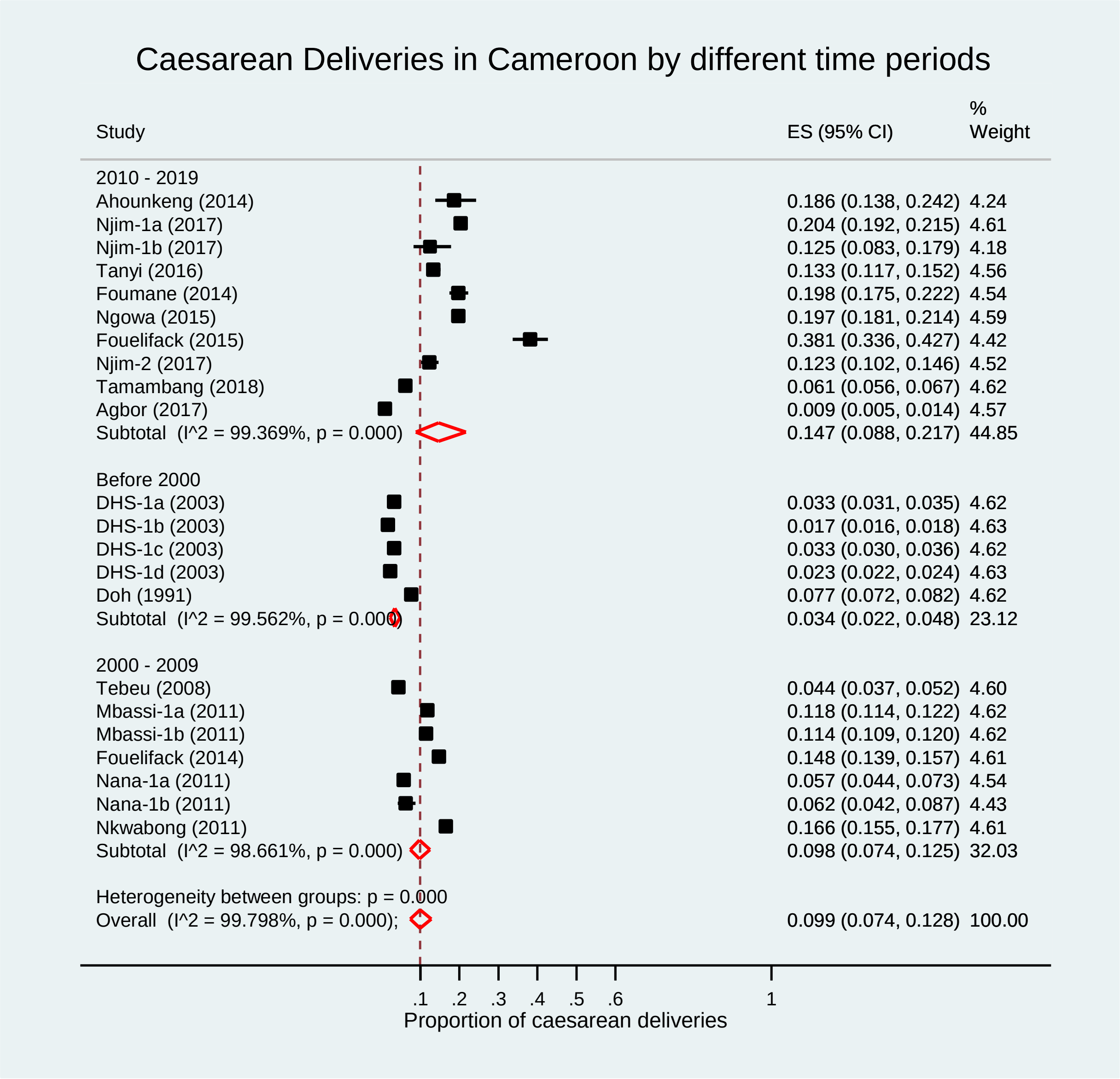

Meta-analysis showing the prevalence of caesarean deliveries in Cameroon in different health facilities – primary, secondary and tertiary.

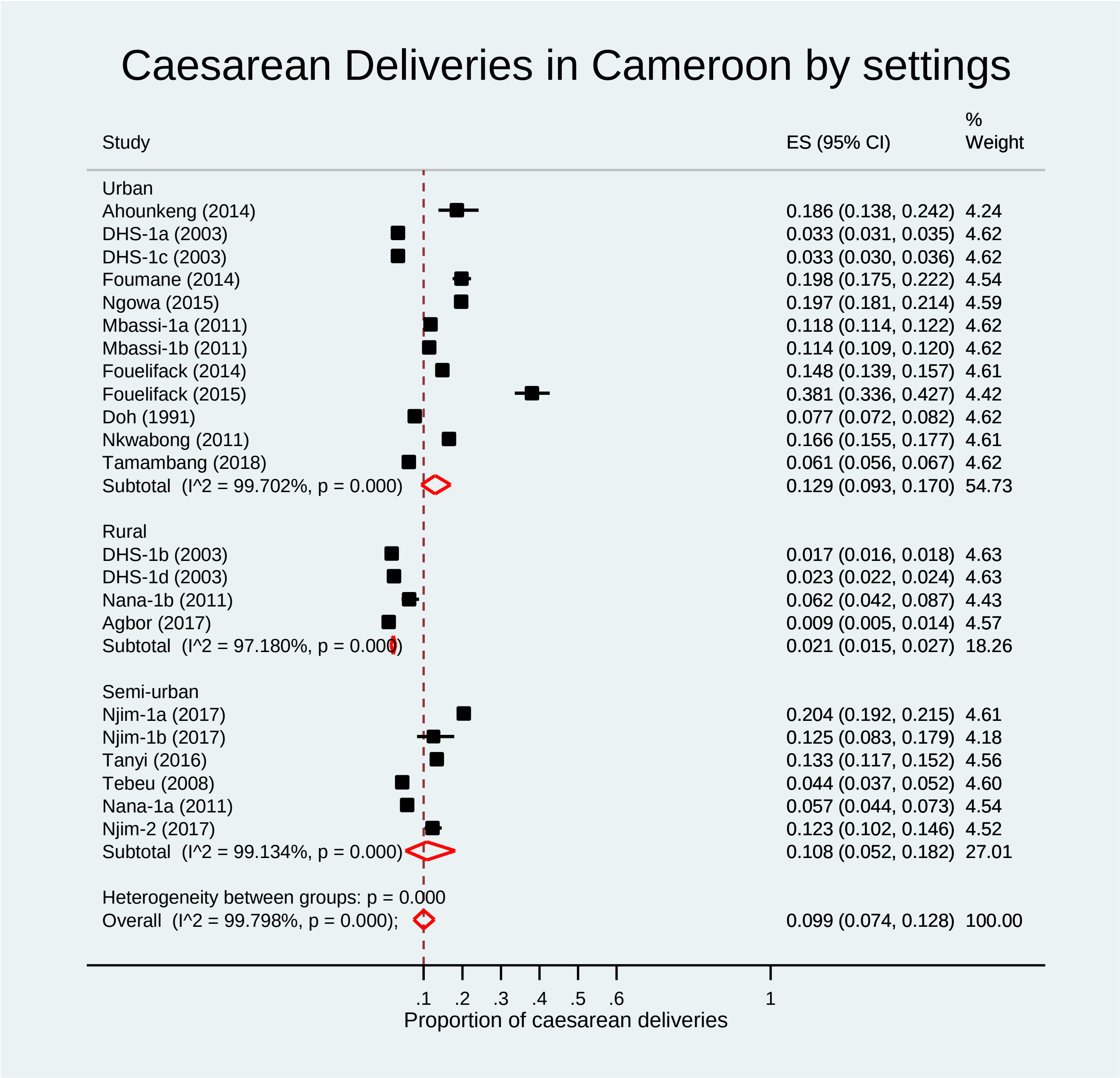

Supplement: Supplementary file 4 — Additional file 4. [file 13690_2020_430_MOESM4_ESM.pdf]
