# Supplementary material for: Prevalence, indications and neonatal complications of caesarean deliveries in Cameroon: a systematic review and meta-analysis
Source: Arch Public Health. 2020 Jun 3;78:51. doi: 10.1186/s13690-020-00430-1 (PMC7268214; doi:10.1186/s13690-020-00430-1)

Meta-analysis showing the proportion of caesarean deliveries due to cephalopelvic disproportions

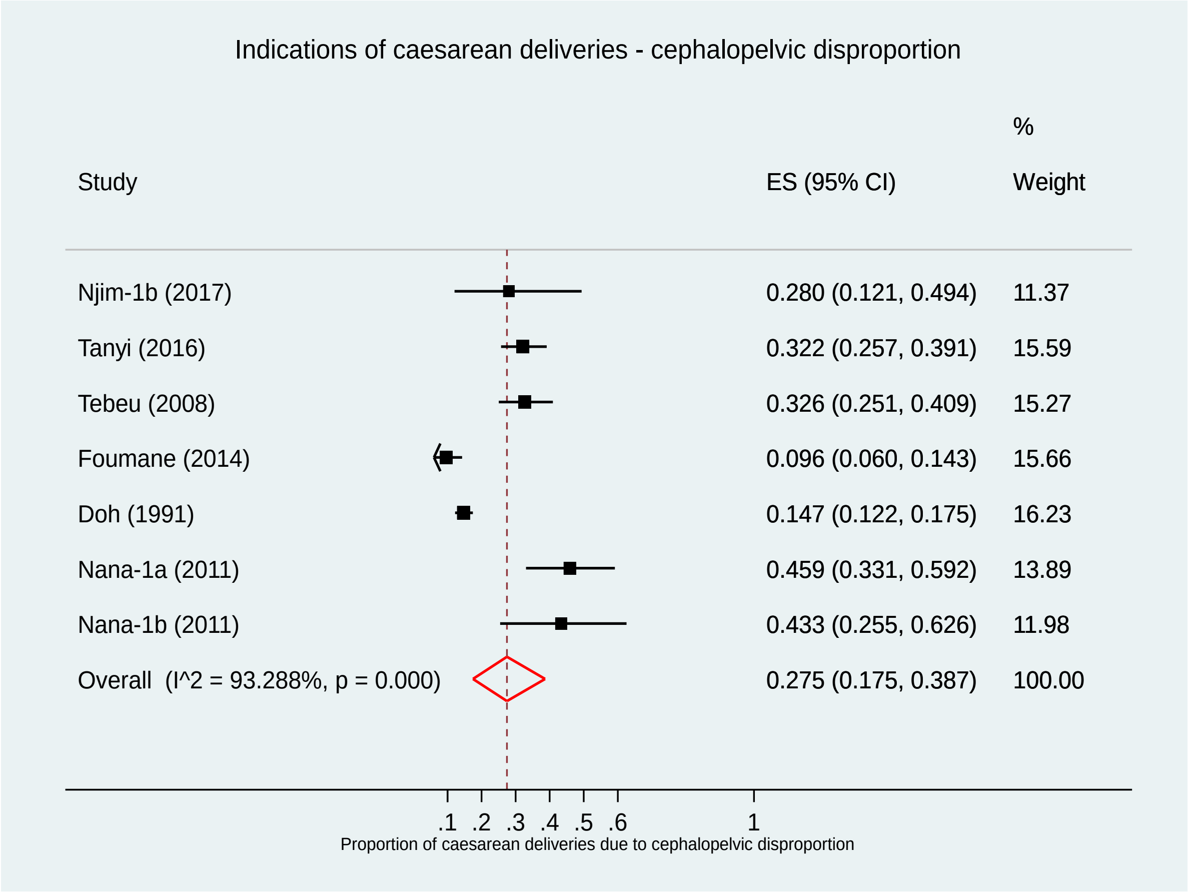

Meta-analysis showing the proportion of caesarean deliveries due to a previous caesarean section

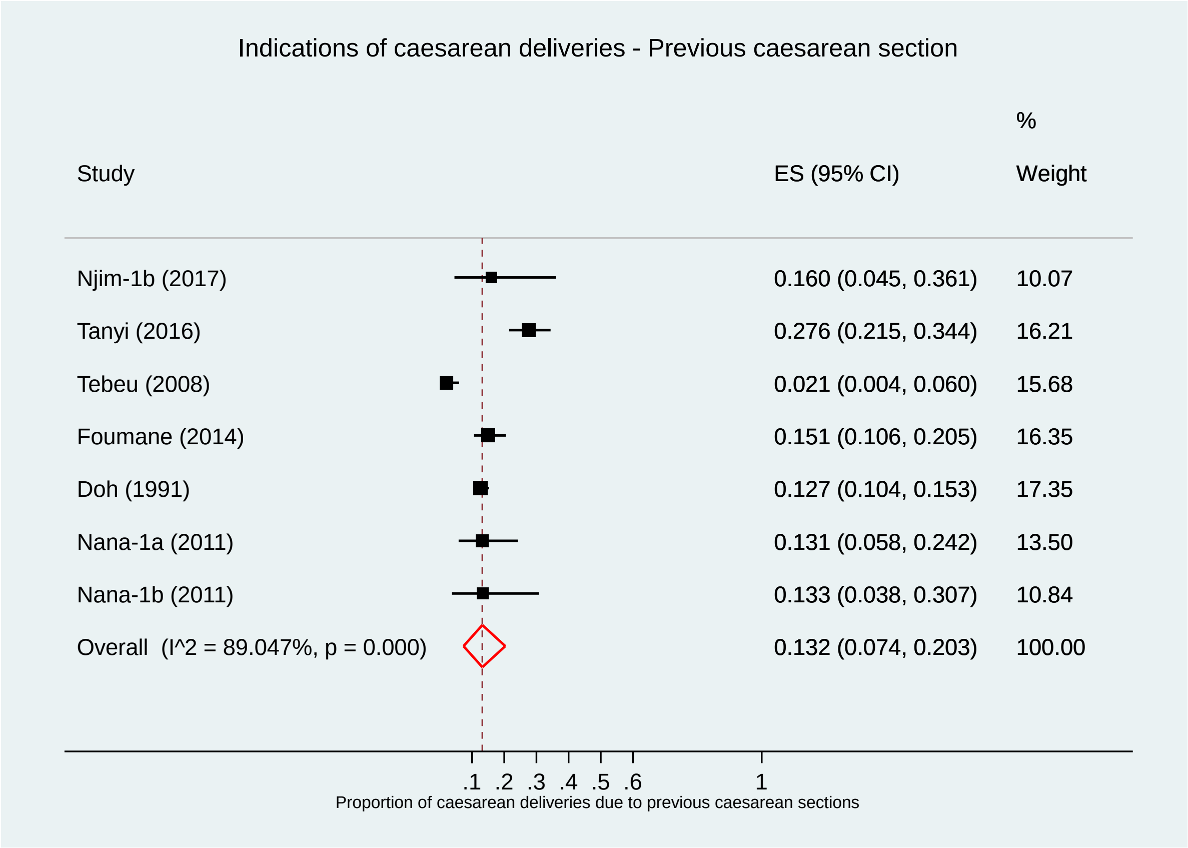

Meta-analysis showing the proportion of caesarean deliveries due to foetal distress

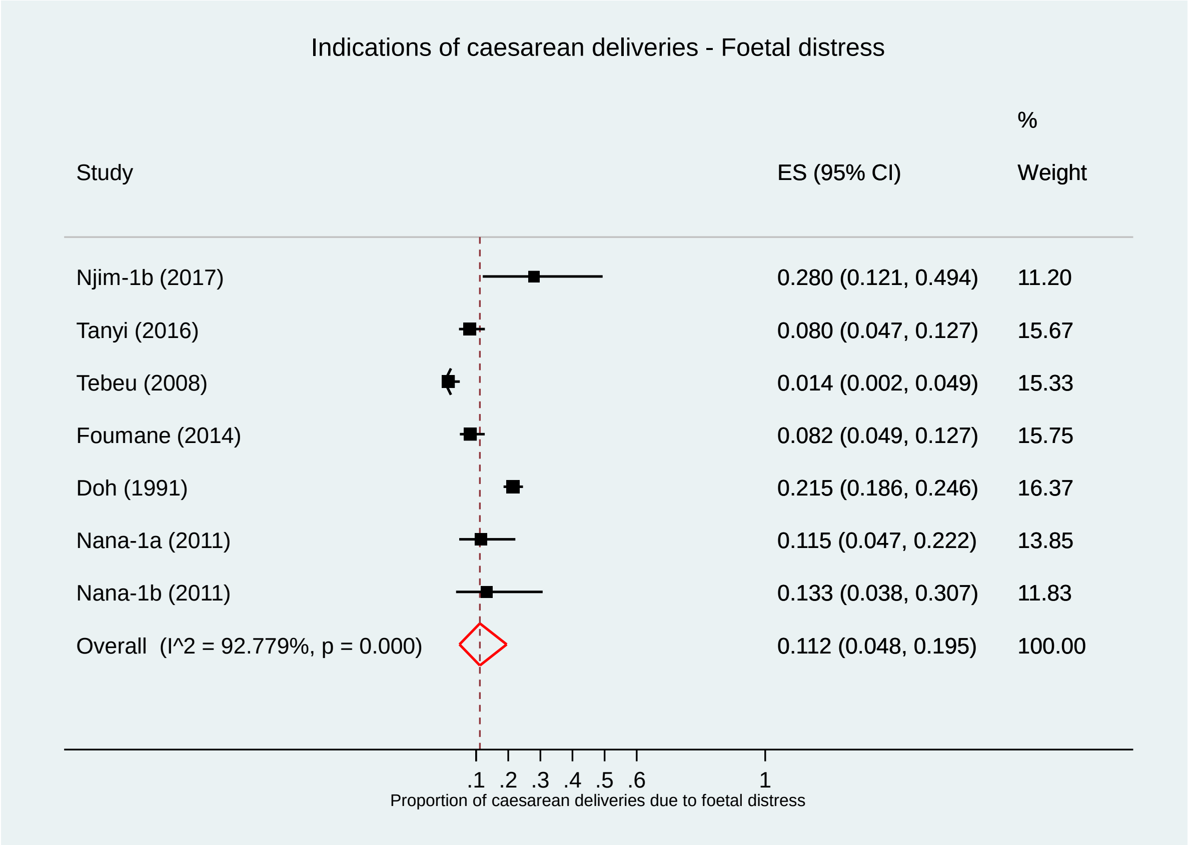

Meta-analysis showing the proportion of caesarean deliveries due to malpresentation

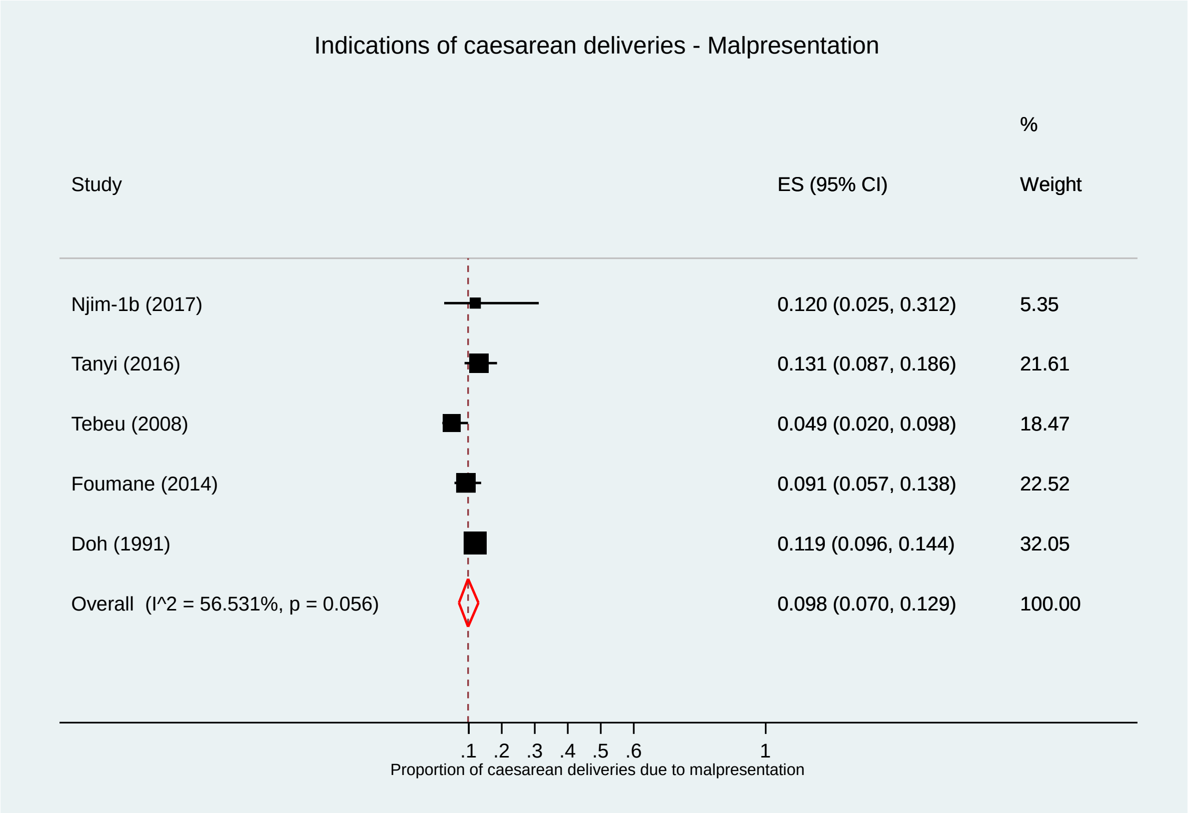

Meta-analysis showing the proportion of caesarean deliveries due to antepartum haemorrhage

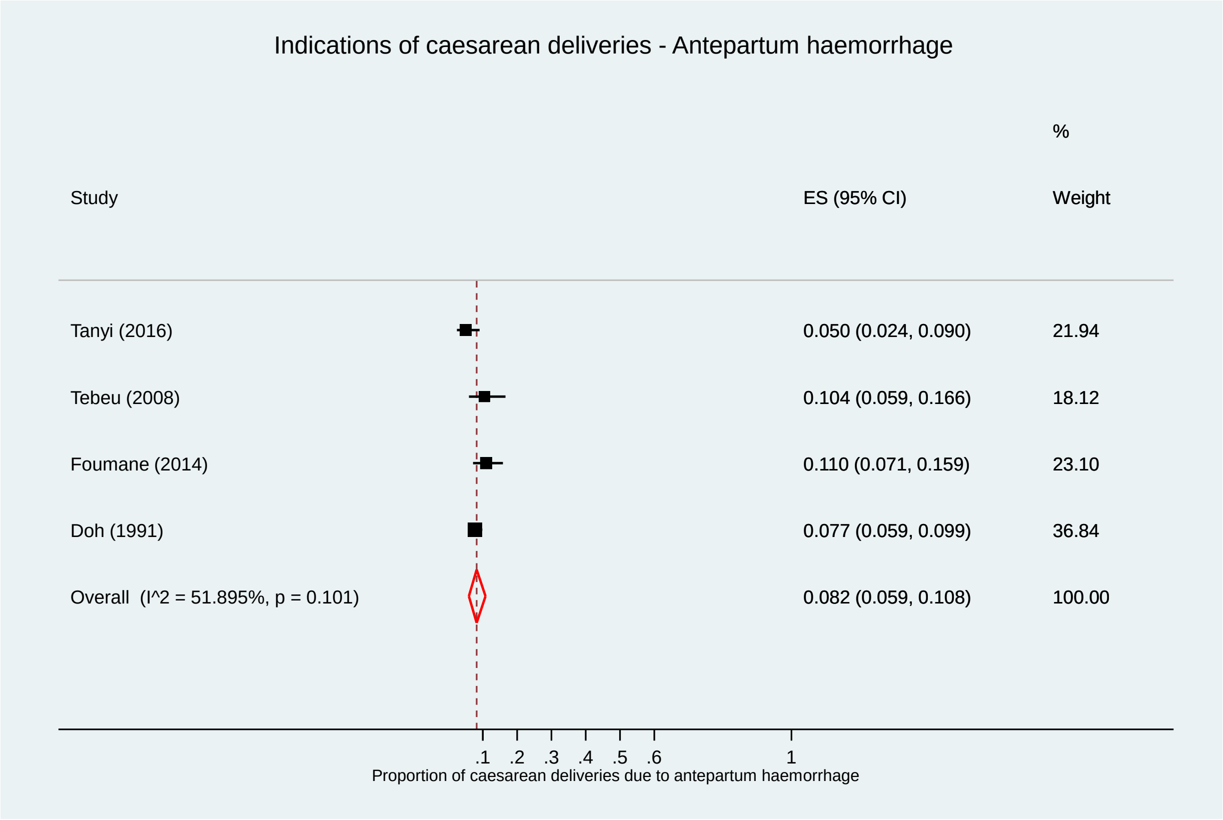

Meta-analysis showing the proportion of caesarean deliveries due to dystocia

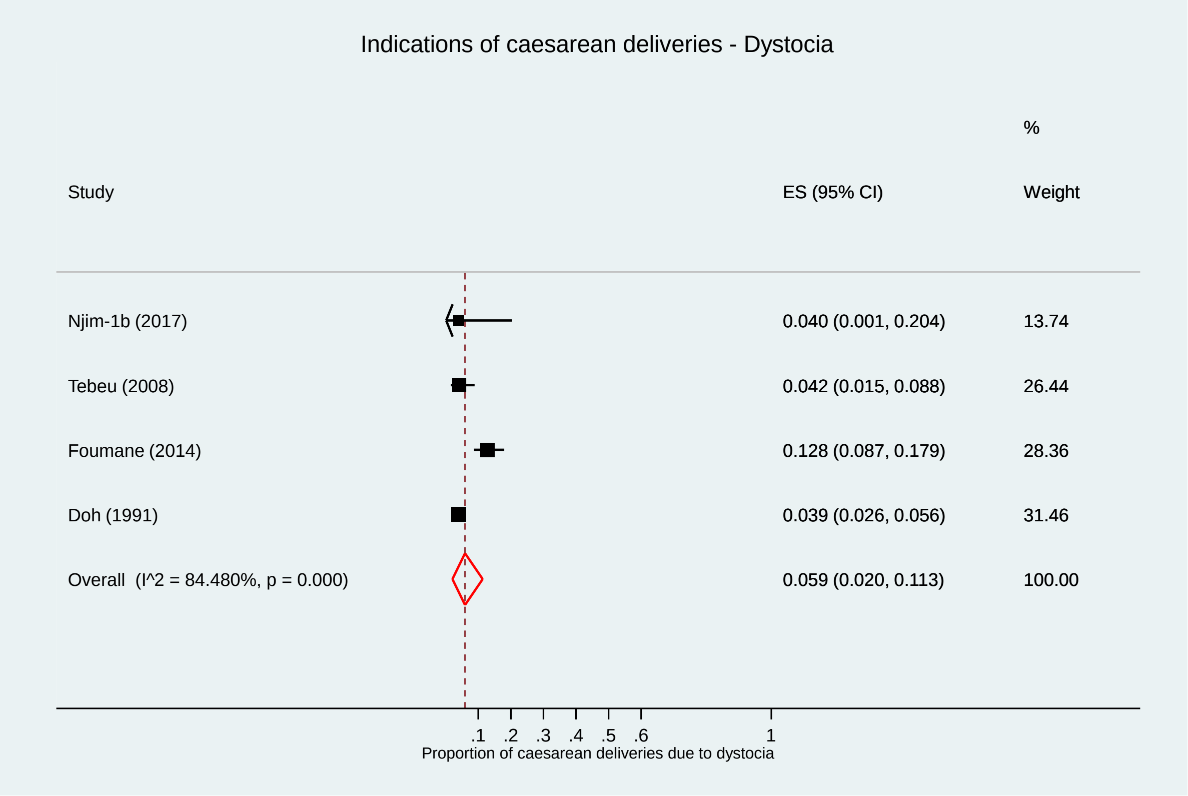

Meta-analysis showing the proportion of caesarean deliveries due to multiple pregnancies

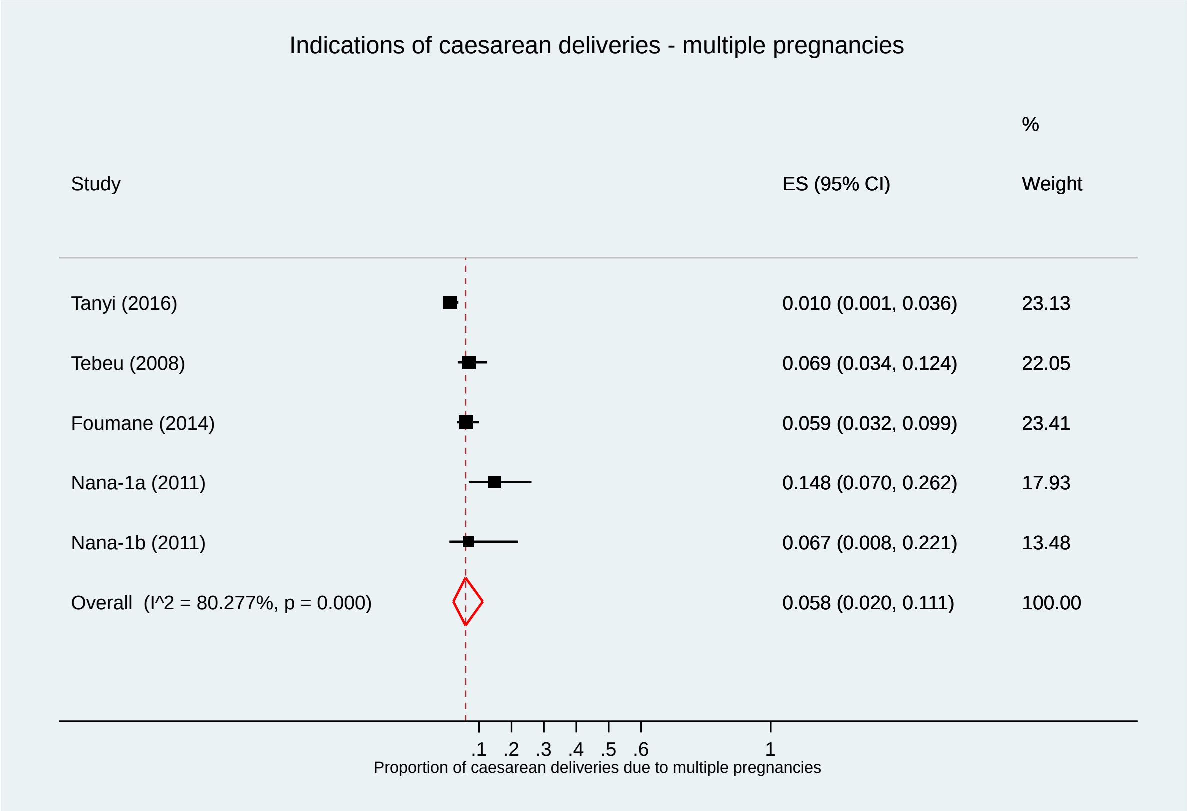

Meta-analysis showing the proportion of caesarean deliveries due to cord prolapse

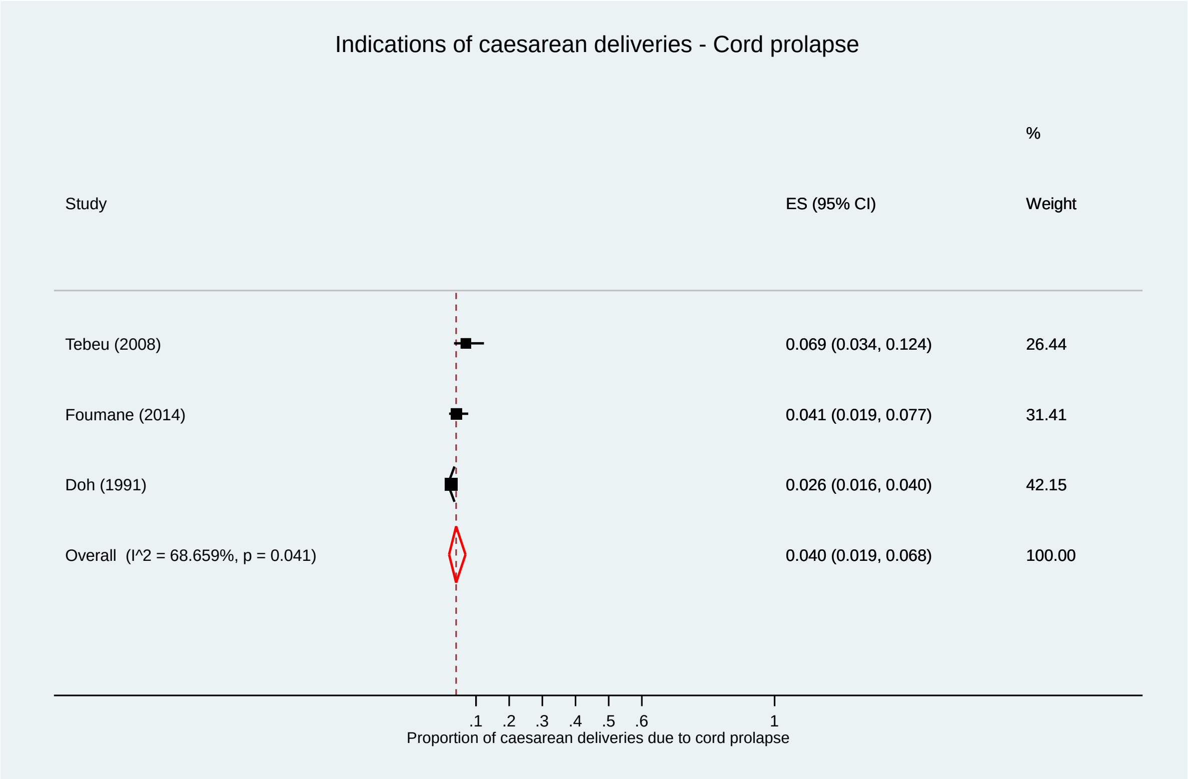

Meta-analysis showing the proportion of caesarean deliveries due to hypertensive disorders in pregnancy

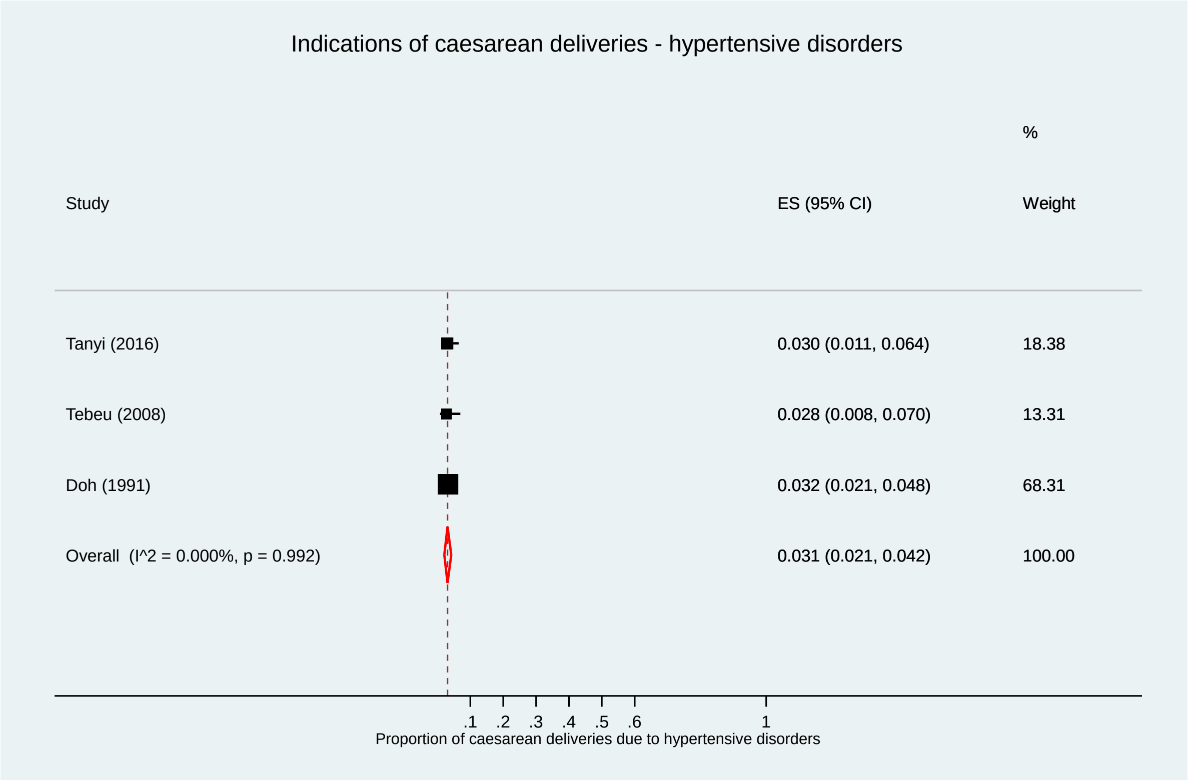

Meta-analysis showing the proportion of caesarean deliveries due to macrosomia

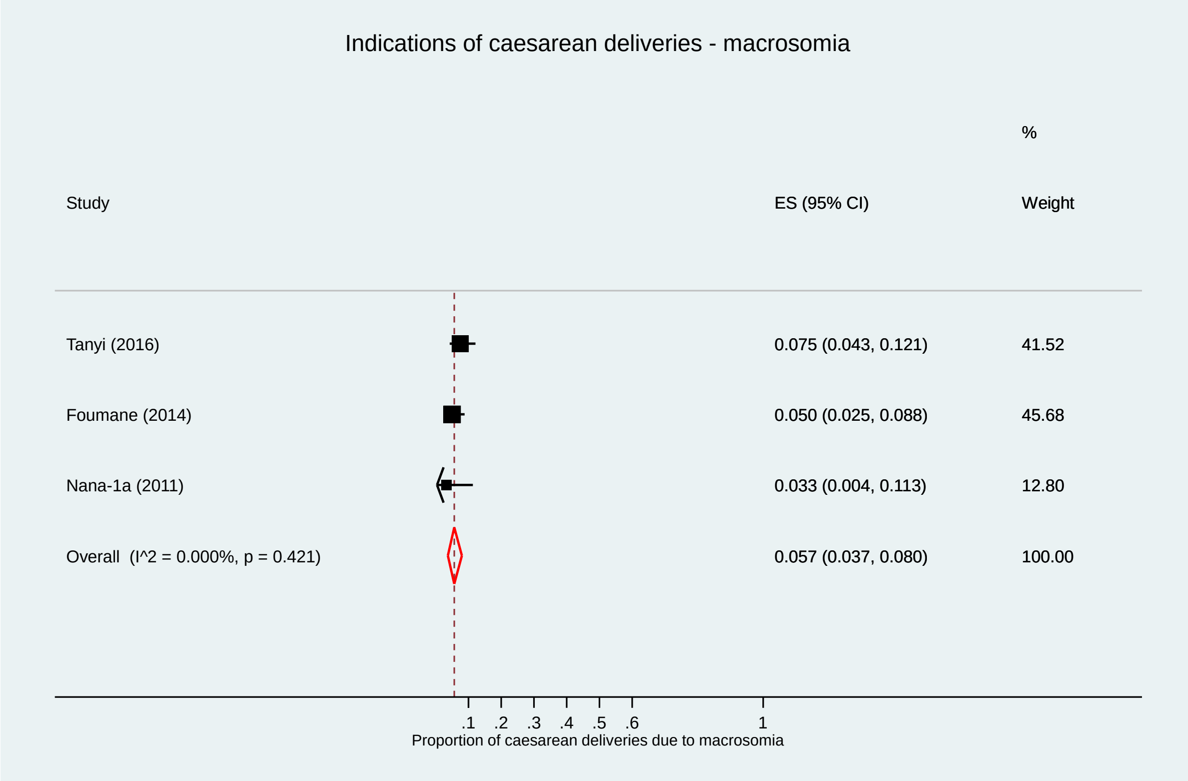

Meta-analysis showing the proportion of caesarean deliveries due to uterine rupture

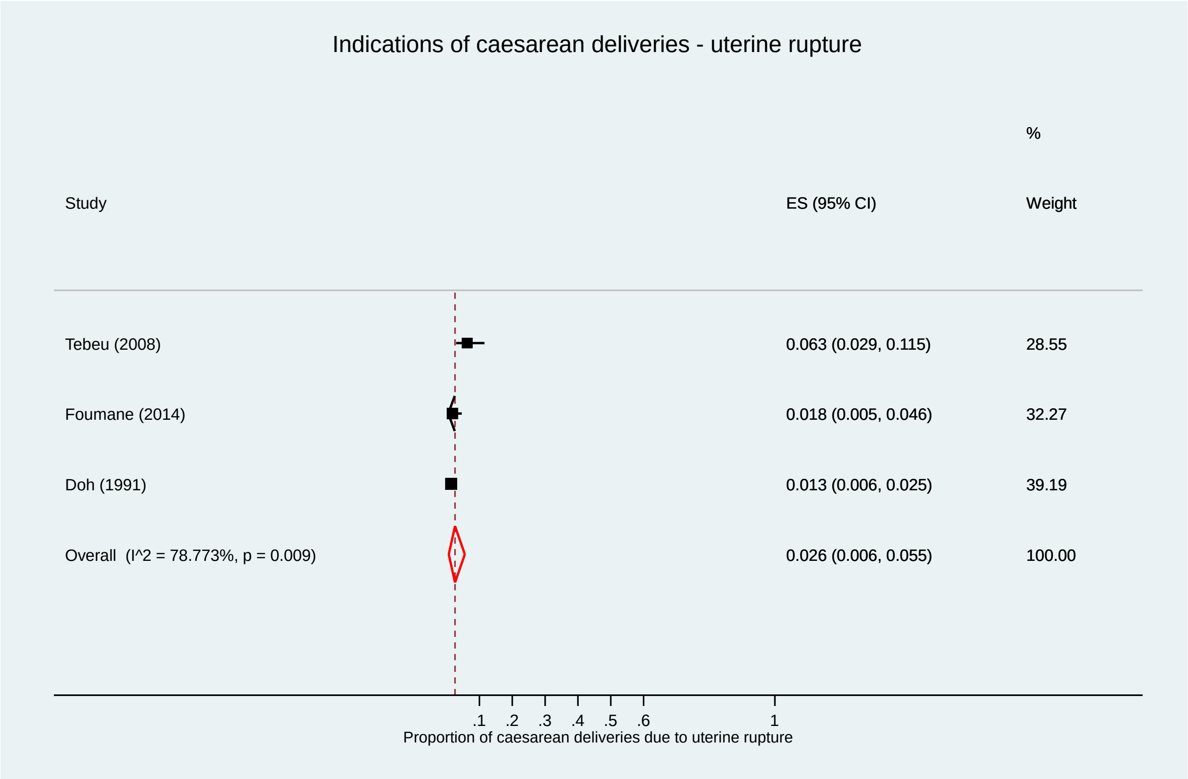

Supplement: Supplementary file 5 — Additional file 5. [file 13690_2020_430_MOESM5_ESM.pdf]
