# Supplementary material for: Prevalence, indications and neonatal complications of caesarean deliveries in Cameroon: a systematic review and meta-analysis
Source: Arch Public Health. 2020 Jun 3;78:51. doi: 10.1186/s13690-020-00430-1 (PMC7268214; doi:10.1186/s13690-020-00430-1)
Supplement: Supplementary file 6 — Additional file 6. [file 13690_2020_430_MOESM6_ESM.pdf]

Meta-analysis showing neonatal asphyxia as a complication of caesarean deliveries in Cameroon

Asphyxia defined as Apgar score of less than 7 in the first minute

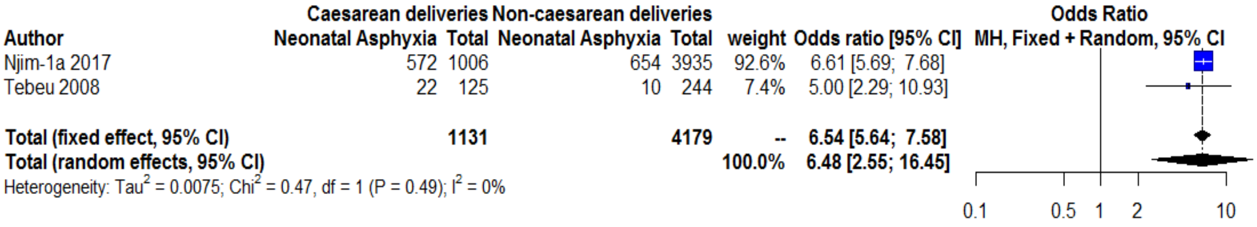

Meta-analysis showing stillbirths as a complication of caesarean deliveries in Cameroon

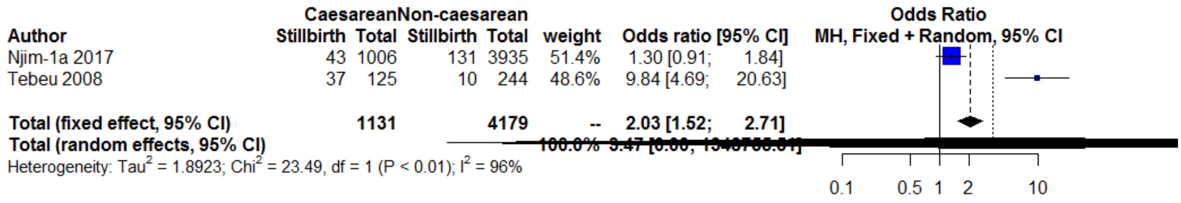

Odds ratio of Random effects model = 3.5
Confidence interval of random effects model: [0.0; 1348755.5]
